# Association between triglyceride-glucose index and atrial fibrillation: A retrospective observational study

**DOI:** 10.3389/fendo.2022.1047927

**Published:** 2022-12-08

**Authors:** Shengnan Chen, Qiao Mei, Li Guo, Xiaoli Yang, Wenbin Luo, Xuemei Qu, Xiaoping Li, Bingqing Zhou, Ken Chen, Chunyu Zeng

**Affiliations:** ^1^ ChongQing Medical University, Chongqing, China; ^2^ Department of Cardiology, Daping Hospital, The Third Military Medical University (Army Medical University), Chongqing, China; ^3^ Cardiovascular Research Center of Chongqing College, Chinese Academy of Sciences, University of Chinese Academy of Sciences, Chongqing, China; ^4^ Department of Endocrinology, Southwest Hospital, The Third Military Medical University (Army Medical University), Chongqing, China; ^5^ Chongqing Key Laboratory for Hypertension Research, Chongqing Cardiovascular Clinical Research Center Chongqing Institute of Cardiology, Chongqing, China; ^6^ Department of Cardiology, The Fifth People’s Hospital of Chongqing, Chongqing, China

**Keywords:** atrial fibrillation, triglyceride-glucose index, insulin resistance, diabet mellitus type 2, arrhythmia

## Abstract

**Background:**

Insulin resistance is associated with atrial remodeling as well as atrial fibrillation (AF). However, there was limited evidence on the relationship of triglyceride-glucose index (TyG) index, a simple, valuable marker of insulin resistance, with AF. Thus, we aimed to investigate the association between TyG index and AF among hospitalized patients.

**Methods:**

A retrospective observational study was conducted in Daping Hospital, which included 356 hospitalized patients from the Department of Cardiology. Clinical and biochemical parameters were collected from electronic medical records and AF was diagnosed from electrocardiogram (ECG) findings.

**Results:**

We found that the TyG index was significantly higher in the AF group than in the group without AF. Multivariate logistic regression revealed that hypertension (OR = 1.756, 95%CI 1.135-2.717, *P* = 0.011) and TyG index (OR = 2.092, 95%CI 1.412-3.100, *P*<0.001) were positively associated with AF. The analysis of the area under the ROC curve was performed and revealed that area under curve (AUC) of TyG index was 0.600 (95%CI, 0.542-0.659, *P* = 0.001), the optimal critical value was 8.35, the sensitivity was 65.4%, and the specificity was 52.0%. Additional subgroup analyses of diabetic and non-diabetic subjects were also performed and found the TyG index was increased in non-diabetic subjects with AF. Furthermore, a logistic regression analysis showed TyG index was associated with AF (OR = 3.065, 95% CI, 1.819-5.166, *P*<0.001) in non-diabetic subjects. However, TyG index was not associated with AF in diabetic subjects.

**Conclusion:**

Elevated TyG index is an independent risk factor for AF among non-diabetic hospitalized patients.

## Introduction

Atrial fibrillation (AF) is a cardiac arrhythmia associated with an elevated risk of stroke, heart failure, and mortality. Previous studies have indicated the fundamental electrophysiological and structural changes within the left atrium are the main pathophysiological basis of AF (1). And increased evidence has revealed the metabolism disorders, especially diabetes and insulin resistance (IR), could lead to the atrial electrical and structural remodeling (1–3), which contributes to the initiation of AF. However, current conclusions are conflicting. Various parameters of insulin sensitivity are used to evaluate the relationship between IR and the prevalence of AF. For example, fasting insulin has generally not been shown to increase the risk of incident AF (4). Other study showed AF development increased at the homeostatic model of insulin resistance (HOMA-IR) levels between 1 to 2.5, whereas no further increase occurred above HOMA-IR as 2.5, indicated a non-linear relationship between IR and AF in non-diabetics (5, 6). Since the calculation of HOMA-IR depends on atypical measurement of serum insulin levels, it has limited application in clinical practice (7). In this sense, triglyceride-glucose (TyG) index has been proposed as an alternative method and valuable marker to evaluate IR.

TyG index was first reported in 2008 by combining fasting triglyceride (TG) levels with fasting plasma glucose (FPG) levels (8). Previous studies showed TyG index has high sensitivity and specificity with a close relationship with HOMA-IR and the total glucose metabolism rates (8–10). Clinical studies have demonstrated that increased TyG index is significantly correlated to higher risk of coronary artery disease and carotid atherosclerosis and greater prevalence of major adverse cardiovascular events in patients with acute coronary syndrome (11–14). Since TyG index has been widely used as a marker of insulin resistance and a predictor of several metabolic and cardiovascular diseases, the purpose of this study was to evaluate the association between TyG index and AF.

## Methods

### Study design and setting

This study was a retrospective, observational study and the clinical data was obtained from electronic medical records. The study was reviewed and approved by the Research Ethics Board in Daping Hospital (No. 181, 2022, Chongqing City, P.R. China).

### Study population

**Figure 1:**
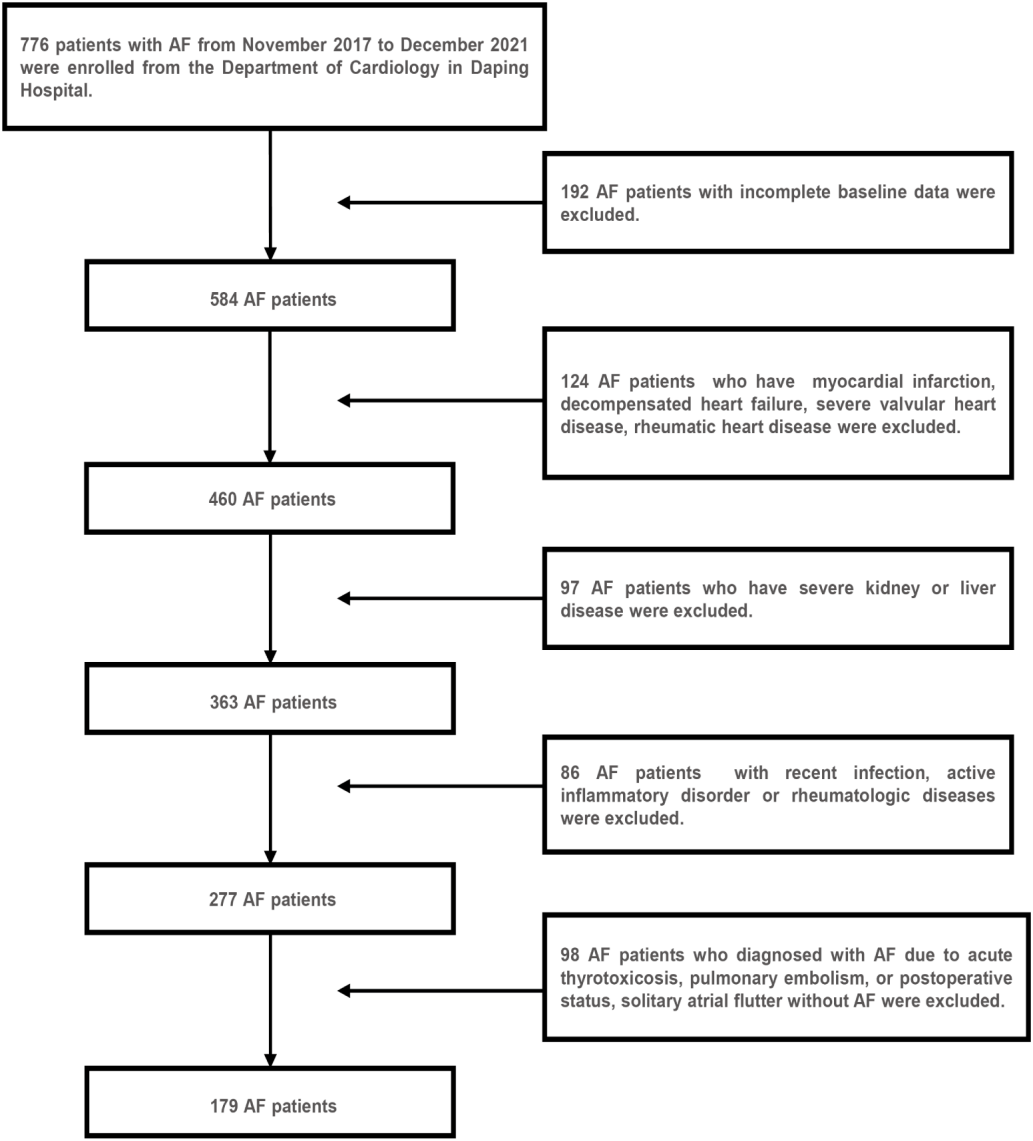
Flow chart of study selection for the AF group.

A total of 179 AF hospitalized patients from the Department of Cardiology in Daping Hospital between November 2017 and December 2021 were enrolled retrospectively in this study, of which 36 patients were combined with diabetes mellitus. **Figure 1** showed the results of the AF patient selection flow chart. AF was diagnosed based on electrocardiograph (ECG) findings (absence of consistent P waves, presence of rapid, irregular f waves with a frequency of 350-600 b.p.m. and an irregular ventricular response) (15). The inclusion criteria were as follows (1): an ECG-confirmed diagnosis of AF (2); aged >18 years (3); complete clinical data. The exclusion criteria are as follows (16–18) (1): structural heart diseases and ischemia induced AF, such as myocardial infarction, decompensated heart failure, severe valvular heart disease, rheumatic heart disease (2); diagnosed with AF due to a reversible cause, such as acute thyrotoxicosis, pulmonary embolism, or postoperative status, solitary atrial flutter without AF (3); previous diagnosis of severe kidney or liver disease, recent infection, active inflammatory disorder or rheumatologic diseases that might influence IR or the TyG index. In addition, 179 age- and gender-matched subjects without AF who were hospitalized in the department during the same time period were randomly sampled and enrolled as the control group. Moreover, the control group included 36 age- and sex-matched diabetic subjects for the subgroup analyses.

### Survey and measurement

Clinical information including age, sex, body mass index, history of smoking and alcohol, history of hypertension, diabetes mellitus, coronary heart disease, and current medication history was collected. Coronary heart disease included acute coronary syndrome and chronic ischemic syndrome diagnosed by previous clinical manifestations, electrocardiogram evaluation and/or coronary angiography (19, 20). Hypertension was defined as systolic blood pressure ≥140 mmHg, diastolic blood pressure ≥90 mmHg, or use of antihypertensive medications (21). Diabetes mellitus was defined as a fasting plasma glucose level ≥ 126 mg/dL, 2-h postload plasma glucose ≥ 200 mg/dL, HbA1c ≥ 6.5%, or history of medical treatment for diabetes (22).

In both groups, 5 mL of fasting venous blood was collected in the early morning of the day after admission, and the plasma was obtained by centrifugation at 3000 rpm for 5 min. Biochemical parameters, including fasting plasma glucose (FPG), total cholesterol (TC), triglycerides (TG), low density lipoprotein cholesterol (LDL-C) and high density lipoprotein cholesterol (HDL-C) were measured using the AU5800 automatic biochemical analyzer (Beckman Coulter, United States). The TyG index was calculated using this formula: ln[fasting triglycerides (mg/dL) × fasting plasma glucose (mg/dL)/2] (23).

### Statistical analysis

Statistical analysis was performed with SPSS version 25.0 (IBM). Quantitative variables if normally distributed were expressed as means ± standard deviation, and t-test was used for the intergroup comparison. Non-normally distributed continuous variables were expressed as median [P25, P75] and Mann–Whitney U test was employed. Categorical data were expressed as percentages, while the differences among groups were compared by Chi square test. The risk of AF was compared using logistic regression analysis. The receiver operating characteristic (ROC) curve was used to determine the optimal cut-off of TyG index for predicting AF and predictive accuracies were quantified and compared using the area under the ROC curve (AUC). All reported probability values were two-tailed, and *P*<0.05 was considered statistically significant.

## Results

### Baseline characteristic

**Figure 2:**
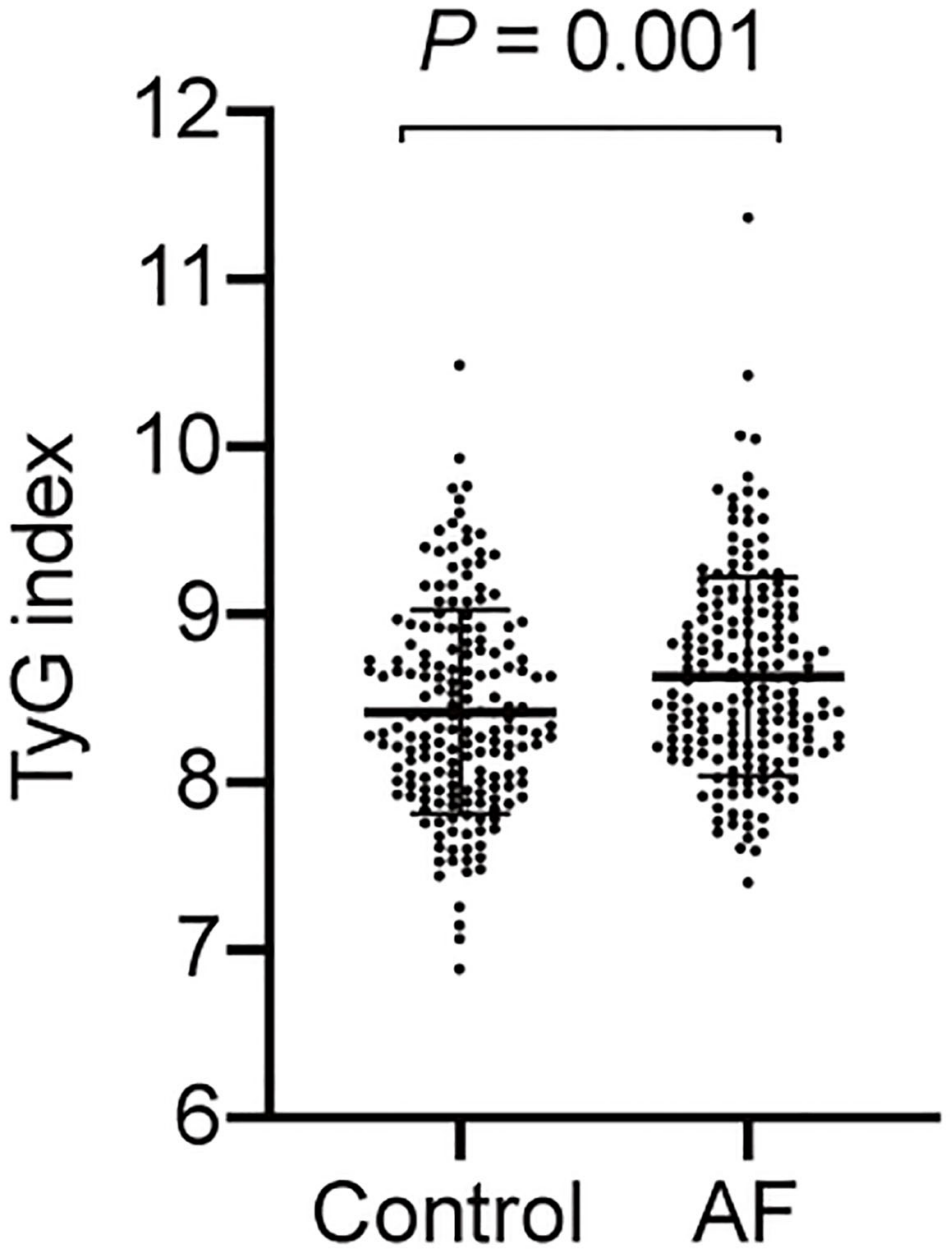
The TyG index in AF group and control (*P* = 0.001).

The baseline characteristics of all participants are summarized in **Table 1**. The levels of BMI, FPG and TG were higher in the AF patients than the participants without AF, whereas the TC level was lower in the controls. And AF patients had a greater proportion of hypertension and history of medication taking. Moreover, the TyG index of the AF group was significantly higher compared to the control group (**Figure 2**).

**Table 1:** Baseline characteristics of all subjects.

Variables	Control n=179	AF n=179	*P*-value
**Demographics**			
Male sex, n (%)	92 (51.4%)	95 (53.1%)	0.751
Age, median (IQR), years	67 (61-73)	68 (61-73)	0.724
Smoking, n (%)	46 (25.7%)	52 (29.2%)	0.457
Drinking, n (%)	35 (19.6%)	42 (23.6%)	0.353
**Comorbid conditions**			
Hypertension, n (%)	78 (43.6%)	109 (60.9%)	0.001
DM, n (%)	36 (20.1%)	36 (20.1%)	1
CAD, n (%)	37 (20.7%)	35 (19.6%)	0.792
**Medication**			
ACEI/ARB, n (%)	8 (4.5%)	33 (18.4%)	<0.001
Beta-blockers, n (%)	6 (3.4%)	29 (16.2%)	<0.001
CCB, n (%)	17 (9.5%)	31 (17.3%)	0.030
Lipid-lowering drugs, n (%)	8 (4.5%)	15 (8.4%)	0.131
**Physical examination**			
BMI, median (IQR), kg/m^2^	23.31 (21.46-25.60)	24.30 (22.51-26.57)	0.001
SBP, median (IQR), mmHg	129 (113-139)	127 (112-143)	0.797
DBP, median (IQR), mmHg	77 (69-85)	78 (70-89)	0.127
**Laboratory data**			
FPG, mmol/L, median (IQR)	4.72 (4.27-5.28)	5.01 (4.52-5.65)	0.004
TC, mmol/L, median (IQR)	4.23 (3.52-4.93)	3.92 (3.37-4.50)	0.007
TG, mmol/L, median (IQR)	1.13 (0.80-1.62)	1.27 (0.93-1.84)	0.010
HDL-C, mmol/L, median (IQR)	1.16 (1.01-1.36)	1.10 (0.95-1.27)	0.011
LDL-C, mmol/L, mean ± SD	2.69 ± 0.76	2.53 ± 0.70	0.039

AF, atrial fibrillation; BMI, body mass index; SD, standard deviation; IQR, interquartile range; DM, diabetes mellitus; CAD, coronary artery disease; ACEI/ARB, angiotensin-converting enzyme inhibitors/angiotensin II receptor blockers; CCB, calcium channel blockers; SBP, systolic blood pressure; DBP, diastolic blood pressure; FPG, fasting plasma glucose; TC, total cholesterol; TG, triglyceride; HDL-C, high-density lipoprotein cholesterol; LDL-C, low-density lipoprotein cholesterol.

### Association of TyG index with AF

Since previous studies have identified male sex, aging, hypertension, diabetes, obesity, smoking and excessive alcohol use are the as independent risk factors (24, 25), a bivariate logistic regression analysis was conducted to identify those candidates for multivariate logistic regression analysis. And the univariate logistic regression analysis indicated BMI, hypertension, TC, TG, HDL-C, LDL-C and TyG index were associated with AF (*P*<0.05 for all) (**Table 2**). All independent variables with a *P*-value of<0.05 in bivariate logistic regression analysis were included in the multivariable logistic regression analysis. This result showed hypertension (OR = 1.756, 95%CI 1.135-2.717, *P* = 0.011) and TyG index (OR = 2.092, 95%CI 1.412-3.100, *P*<0.001) were positively associated with AF (**Table 3**).

**Table 2:** Univariate logistic regression analysis of risk factors for AF in all subjects.

Variables	β	SE	Waldχ2	*P*-value	OR	95% CI
Male sex	0.067	0.212	0.101	0.751	1.069	0.706-1.619
Age	0.003	0.011	0.087	0.769	1.003	0.981-1.026
BMI	0.115	0.035	10.445	0.001	1.121	1.046-1.202
Smoking	0.177	0.238	0.553	0.457	1.193	0.749-1.901
Drinking	0.239	0.258	0.860	0.354	1.271	0.766-2.108
Hypertension	0.701	0.215	10.649	0.001	2.016	1.323-3.072
DM	0	0.264	0	1	1	0.596-1.677
CAD	-0.070	0.264	0.070	0.792	0.933	0.556-1.564
FPG	0.134	0.079	2.836	0.092	1.143	0.978-1.336
TC	-0.290	0.109	7.058	0.008	0.749	0.605-0.927
TG	0.248	0.125	3.930	0.047	1.281	1.003-1.636
HDL-C	-1.289	0.426	9.143	0.002	0.275	0.119-0.635
LDL-C	-0.302	0.147	4.209	0.040	0.739	0.554-0.987
TyG index	0.597	0.184	10.517	0.001	1.817	1.267-2.607

BMI, body mass index; DM, diabetes mellitus; CAD, coronary artery disease; FPG, fasting plasma glucose; TC, total cholesterol; TG, triglyceride; HDL-C, high-density lipoprotein cholesterol; LDL-C, low-density lipoprotein cholesterol; TyG index, triglyceride-glucose index; SE, standard error; OR, odds-ratio; CI, confidence interval.

**Table 3:** Multivariate logistic regression analysis of risk factors for AF in all subjects.

Variables	β	SE	Waldχ2	*P*-value	OR	95% CI
Hypertension	0.563	0.223	6.390	0.011	1.756	1.135-2.717
TC	-0.415	0.120	11.877	0.001	0.661	0.522-0.836
TyG index	0.738	0.201	13.545	<0.001	2.092	1.412-3.100

TC, total cholesterol; TyG index, triglyceride-glucose index; SE, standard error; OR, odds-ratio; CI, confidence interval.

### The TyG index for predicting AF

**Figure 3:**
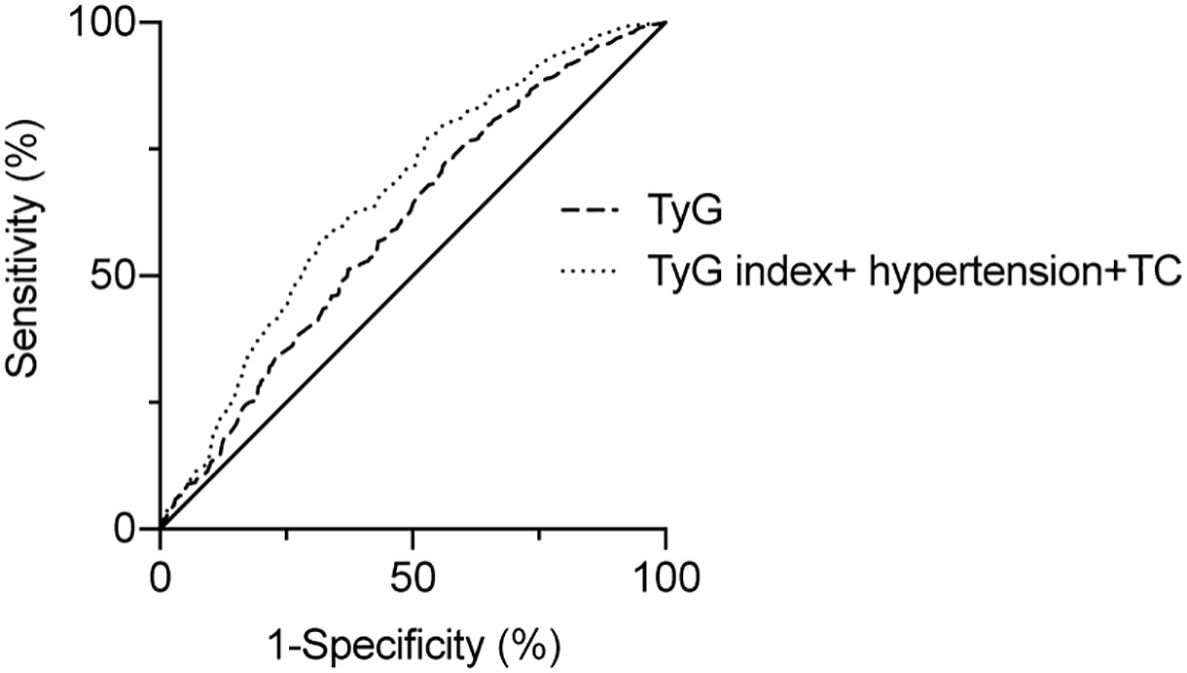
ROC curve of the risk AF according to TyG index.

To assess the predictive value of TyG index for AF, the analysis of the area under the ROC curve was performed and revealed that area under curve (AUC) of TyG index was 0.600 (95%CI, 0.542-0.659, *P* = 0.001), the optimal critical value was 8.35, the sensitivity was 65.4%, and the specificity was 52.0% (**Table 4** and **Figure 3**). A logistic regression model was established based on the results of the multivariate analysis: logistic (P) = -4.873 + 0.563×hypertension – 0.415×TC + 0.738×TyG index. The AUC of the model for predicting AF was 0.667 (95%CI, 0.611-0.723, *P* < 0.001), the cut-off value was 0.466, the sensitivity was 71.5% and the specificity was 58.1%.

**Table 4:** Areas under the receiver operating characteristic curve (AUC) for AF in all subjects.

Variables	AUC	SE	*P*-value	95% CI
TyG index	0.600	0.030	0.001	0.542-0.659
TyG index + hypertension + TC	0.667	0.029	<0.001	0.611-0.723

TC, total cholesterol; TyG index, triglyceride-glucose index; AUC, area under the curve; SE, standard error; OR, odds-ratio; CI, confidence interval.

### Association of TyG index with AF in diabetic and non-diabetic subjects

**Figure 4:**
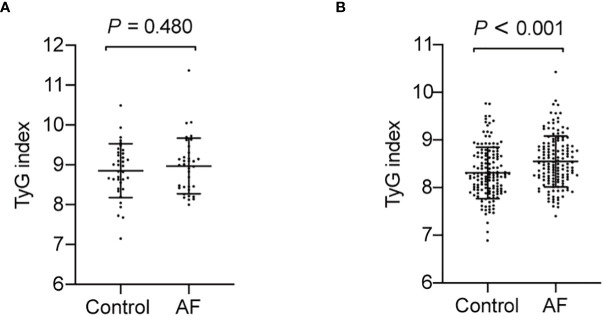
The TyG index in AF group and control in diabetic and non-diabetic subjects. **(A)** The TyG index is no significantly difference in diabetic subjects (*P* = 0.480). **(B)** The TyG index is increased in non-diabetic subjects with AF (*P* < 0.001).

Since previous studies show different result of the causal association between diabetes and AF in diabetic and non-diabetic subjects, we then analyzed the TyG index and AF in diabetic and non-diabetic subjects. The baseline characteristics of diabetic and non-diabetic subjects are summarized in **Supplemental Tables S1**, **S2**. And we found the TyG index is increased in non-diabetic subjects with AF, whereas there is no significantly difference between control and AF group in TyG index in diabetic subjects (**Figure 4**). Furthermore, a logistic regression analysis showed TyG index is associated with AF (OR = 3.065, 95% CI, 1.819-5.166, *P*<0.001) in non-diabetic subjects (**Table 5**). However, TyG index is not associated with AF in diabetic subjects (**Supplemental Table S3**). In non-diabetic subjects, the analysis of the area under the ROC curve showed the AUC was 0.625 (95%CI, 0.560-0.689, *P*<0.001) and the cut-off value was 8.34, the sensitivity was 61.5%, and the specificity was 60.1% (**Supplemental Table S4**). A logistic regression model was established based on the results of the multivariate analysis: logistic (P) = -7.558 + 0.758×hypertension –0.537×TC + 1.120×TyG index. The AUC of the model for predicting AF was 0.704 (95%CI, 0.644-0.764, *P* < 0.001), the cut-off value was 0.506, the sensitivity was 65.7% and the specificity was 70.6%.

**Table 5:** Multivariate logistic regression analysis of risk factors for AF in non-diabetic subjects.

Variables	β	SE	Waldχ2	*P*-value	OR	95% CI
Hypertension	0.758	0.254	8.900	0.003	2.134	1.297-3.510
TC	-0.537	0.153	12.277	<0.001	0.585	0.433-0.789
TyG index	1.120	0.266	17.685	<0.001	3.065	1.819-5.166

TC, total cholesterol; TyG index, triglyceride-glucose index; SE, standard error; OR, odds-ratio; CI, confidence interval.

## Discussion

Although numerous clinical and experimental studies show metabolism disorder is an important risk factor for the development of AF, whether diabetes and its related phenotypes (e.g., high FBG, insulin resistance) can contribute to AF development remains controversial. Our present study revealed that IR might be associated with AF. In this study, we found that the TyG index levels, a practical IR indicator, were higher in AF patients than the controls. Further logistic analysis indicated that the TyG index was an independent predictor for AF after adjusting for other factors. And the ROC curve confirmed the predictive value of the TyG index for AF.

Our present study suggested that IR is an independent predictor of AF, which has been demonstrated in previous cohort and experimental studies. Lee et al. found the baseline IR contributes to the long-term risk of incident AF in a community-based non-diabetes population (6). And a retrospective study of non-diabetic patients who have undergone radiofrequency catheter ablation (RFCA) showed the elevated TyG index was associated with an increased risk of late AF recurrence (26). Data from animal experiment also shows the IR mice were highly vulnerable to AF due to impaired glucose transport in the atria (27). And IR increases the levels of CaMKIIδ oxidizing and phospholamban/RyR-2 phosphorylation, which resulting in abnormal intracellular calcium homeostasis and the atrial structural remodeling (28). However, controversy has emerged regarding the association between IR and AF in various clinical studies. A study including a total of 60,620 individuals with atrial fibrillation with 970,216 control individuals did not find the causal association between diabetes and AF (29). The data from the Framingham Heart Study cannot indicate the association between IR and incident AF (30).

Some potential factors may explain those controversial outcomes. One possible explanation is that the correlation among known AF risk factor, such as hypertension, BMI and age, could obscure the individual contributions of specific risk factors to incident AF (30, 31). We also noticed that a variety of IR parameter in different studies has been proposed, which might lead to the bias of results. Fasting insulin level has been debated for a reliable IR parameter, for example, fasting insulin could not predict the risk of cardiovascular disease, such as coronary heart disease and stroke (31, 32). Thus, some indices are based on fasting plasma concentrations of glucose and insulin to substitute the fasting insulin level, such as the homeostasis model of insulin resistance (HOMA-IR), the quantitative insulin sensitivity check index (QUICKI) and the fasting insulin resistance index (FIRI) (33, 34). Those surrogate indices have been compared with hyperinsulinemic-euglycemic clamp, the gold standard of IR. In present study, we used TyG index, a simple and easy-to-calculate marker, which has been showed to be significantly correlated with IR measured by the hyperinsulinaemic-euglycaemic clamp test (35, 36). Our study found that hospitalized patients with elevated TyG index had a higher prevalence of AF and that after adjusting for traditional risk factors for AF, the TyG index remained an independent risk factor for AF. Moreover, since different cohorts may lead to the controversial results, an additional subgroup analysis of our study compared the difference between diabetic and non-diabetic population. Similar to what published researches reported (26, 29, 30), the present study showed the elevated TyG index was associated with AF in non-diabetic, whereas a role between TyG index with AF has not been observed in diabetic subjects. Lee et al. have identified the non-linear relationship between IR and the risk of AF, and the AF development increased at the HOMA-IR levels approximately between 1-2.5, and then plateaued afterwards (6). Therefore, the higher level of insulin resistance in patients with diabetes, as higher HOMA-IR or TyG index, might not associate with the risk of AF.

In our study, we showed the TC and LDL-C levels in AF group are significantly lower than the control. The lipid metabolism disorder might play a key role in the pathogenesis of AF (37). However, previous studies have identified higher levels of TC and LDL-C are associated with a lower incidence of AF (38, 39). The underlying mechanisms still remain largely unknown. Lower level of cholesterol could impair calcium handling, adrenergic signaling and the myofibril structure in cardiomyocytes (40), which might be involved in the development of AF.

Our study has several limitations. The current study is a cross-sectional clinical study. A causal correlation of TyG index with AF cannot be proposed due to the research design, which requires further prospective cohort researches for validation. Besides, we noticed that the AUC value in our study shows a mild-to-moderate significance. Although the range of AUC (0.6–0.7) is considered to be clinically useful (41–43), a study with a large sample size is needed to further verify the conclusion and to analyze the value of the TyG index in the evaluation of the progression and prognosis of AF.

In conclusion, our study found that TyG index was an independent risk factor of AF in non-diabetic subjects, which is expected to become a simple and practical biological indicator for predicting the occurrence of AF.

## Data Availability

The original contributions presented in the study are included in the article/**Supplementary Material**. Further inquiries can be directed to the corresponding authors.
