# Supplementary material for: Association between triglyceride-glucose index and atrial fibrillation: A retrospective observational study
Source: Front Endocrinol (Lausanne). 2022 Dec 8;13:1047927. doi: 10.3389/fendo.2022.1047927 (PMC9773201; doi:10.3389/fendo.2022.1047927)
Supplement: Supplementary file 1 [file Table_1.docx]

**Supplemental** **Table S1.** Baseline characteristics of the patients with diabetes.

| **Variables** | Control  n=36 | AF  n=36 | *P*-value |
| --- | --- | --- | --- |
| **Demographics** |  |  |  |
| Male sex, n (%) | 18 (50.0%) | 19 (52.8%) | 0.814 |
| Age, years, mean ± SD | 66.44 ± 9.42 | 68.44 ± 8.92 | 0.358 |
| BMI, kg/m^2^, mean ± SD | 25.13 ± 2.87 | 26.15 ± 2.81 | 0.130 |
| Smoking, n (%) | 8 (22.2%) | 4 (11.1%) | 0.206 |
| Drinking, n (%) | 6 (16.7%) | 6 (16.7%) | 1.000 |
| **Comorbid conditions** |  |  |  |
| Hypertension, n (%) | 27 (75.0%) | 27 (75.0%) | 1.000 |
| CAD, n (%) | 16 (44.4%) | 13 (36.1%) | 0.471 |
| **Medication** |  |  |  |
| ACEI/ARB, n (%) | 6 (16.7%) | 13 (36.1%) | 0.061 |
| Beta-blockers, n (%) | 2 (5.6%) | 12 (33.3%) | 0.003 |
| CCB, n (%) | 6 (16.7%) | 8 (22.2%) | 0.551 |
| **Physical examination** |  |  |  |
| SBP, mmHg, mean ± SD | 133.69±15.93 | 131.19 ± 19.00 | 0.547 |
| DBP, mmHg, mean ± SD | 78.94 ± 10.25 | 77.08 ± 10.76 | 0.455 |
| **Laboratory data** |  |  |  |
| FPG, mmol/L, median (IQR) | 6.36 (4.79-8.17) | 6.25 (5.40-8.04) | 0.585 |
| TC, mmol/L, mean ± SD | 4.18 ± 1.17 | 3.81 ± 1.13 | 0.182 |
| TG, mmol/L, median (IQR) | 1.55 (1.00-1.94) | 1.44 (1.06-2.03） | 0.761 |
| HDL-C, mmol/L, mean ± SD | 1.13 ± 0.19 | 1.05 ± 0.25 | 0.122 |
| LDL-C, mmol/L, mean ± SD | 2.63 ± 0.85 | 2.38 ± 0.83 | 0.206 |

**Abbreviations:** AF: atrial fibrillation; BMI: body mass index; SD: standard deviation; IQR: interquartile range; CAD: coronary artery disease; ACEI/ARB: angiotensin- converting enzyme inhibitors/angiotensin II receptor blockers; CCB: calcium channel blockers; SBP: systolic blood pressure; DBP: diastolic blood pressure; FPG: fasting plasma glucose; TC: total cholesterol; TG: triglyceride; HDL-C: high-density lipoprotein cholesterol; LDL-C: low-density lipoprotein cholesterol.

**Supplemental** **Table S2.** Baseline characteristics of the patients without diabetes.

| **Variables** | Control  n=143 | AF  n=143 | *P*-value |
| --- | --- | --- | --- |
| **Demographics** |  |  |  |
| Male sex, n (%) | 74 (51.7%) | 76 (53.1%) | 0.813 |
| Age, median (IQR), y | 68 (61-73) | 67 (61-72) | 0.881 |
| BMI, median (IQR), kg/m^2^, | 22.90 (21.16-24.98) | 24.00 (22.31-25.88) | 0.001 |
| Smoking, n (%) | 38 (26.6%) | 48 (33.8%) | 0.184 |
| Drinking, n (%) | 29 (20.3%) | 36 (25.4%) | 0.308 |
| **Comorbid conditions** |  |  |  |
| Hypertension, n (%) | 51 (35.7%) | 82 (57.3%) | ＜0.001 |
| CAD, n (%) | 21 (14.7%) | 22 (15.4%) | 0.869 |
| **Medication** |  |  |  |
| ACEI/ARB, n (%) | 2 (1.4%) | 20 (14.0%) | ＜0.001 |
| Beta-blockers, n (%) | 4 (2.8%) | 17 (11.9%) | 0.003 |
| CCB, n (%) | 11 (7.7%) | 23 (16.1%) | 0.028 |
| **Physical examination** |  |  |  |
| SBP, mmHg, median (IQR) | 127 (112-139) | 126 (112-141) | 0.999 |
| DBP, mmHg, mean ± SD | 77.19 ± 12.63 | 80.24 ± 13.74 | 0.051 |
| **Laboratory data** |  |  |  |
| FPG, mmol/L, median (IQR) | 4.61 (4.23-5.02) | 4.83 (4.44-5.37) | 0.003 |
| TC, mmol/L, mean ± SD | 4.30 ± 1.00 | 4.03 ± 0.90 | 0.020 |
| TG, mmol/L, median (IQR) | 1.04 (0.78-1.49) | 1.24 (0.92-1.76) | 0.004 |
| HDL-C, mmol/L, median (IQR) | 1.16 (1.01-1.38) | 1.12 (0.98-1.27) | 0.035 |
| LDL-C, mmol/L, mean ± SD | 2.70 ± 0.73 | 2.57 ± 0.66 | 0.101 |

**Abbreviations:** AF: atrial fibrillation; BMI: body mass index; SD: standard deviation; IQR: interquartile range; CAD: coronary artery disease; ACEI/ARB: angiotensin- converting enzyme inhibitors/angiotensin II receptor blockers; CCB: calcium channel blockers; SBP: systolic blood pressure; DBP: diastolic blood pressure; FPG: fasting plasma glucose; TC: total cholesterol; TG: triglyceride; HDL-C: high-density lipoprotein cholesterol; LDL-C: low-density lipoprotein cholesterol.

**Supplemental** **Table S3.** Univariate logistic regression analysis of risk factors for AF

in diabetic subjects.

| **Variables** | β | SE | Waldχ2 | *P*-value | OR | 95% CI |
| --- | --- | --- | --- | --- | --- | --- |
| Male sex | 0.111 | 0.472 | 0.056 | 0.814 | 1.118 | 0.443-2.818 |
| Age | 0.024 | 0.026 | 0.857 | 0.355 | 1.025 | 0.973-1.079 |
| BMI | 0.131 | 0.087 | 2.271 | 0.132 | 1.139 | 0.962-1.350 |
| Smoking | -0.827 | 0.665 | 1.546 | 0.214 | 0.438 | 0.119-1.610 |
| Drinking | 0 | 0.632 | 0 | 1 | 1 | 0.290-3.454 |
| Hypertension | 0 | 0.544 | 0 | 1 | 1 | 0.344-2.906 |
| CAD | -0.347 | 0.483 | 0.518 | 0.472 | 0.707 | 0.274-1.819 |
| FPG | 0.052 | 0.112 | 0.218 | 0.641 | 1.054 | 0.846-1.312 |
| TC | -0.282 | 0.212 | 1.781 | 0.182 | 0.754 | 0.498-1.142 |
| TG | -0.018 | 0.200 | 0.008 | 0.928 | 0.982 | 0.664-1.454 |
| HDL-C | -1.675 | 1.087 | 2.376 | 0.123 | 0.187 | 0.022-1.576 |
| LDL-C | -0.366 | 0.288 | 1.610 | 0.204 | 0.694 | 0.394-1.220 |
| TyG index | 0.251 | 0.352 | 0.509 | 0.475 | 1.286 | 0.645-2.565 |

**Abbreviations:** BMI: body mass index; CAD: coronary artery disease; FPG: fasting plasma glucose; TC: total cholesterol; TG: triglyceride; HDL-C: high-density lipoprotein cholesterol; LDL-C: low-density lipoprotein cholesterol; TyG index: triglyceride-glucose index; SE: standard error; OR: odds-ratio; CI: confidence interval.

**Supplemental** **Table S4.** Areas under the receiver operating characteristic curve (AUC) for AF in non-diabetic subjects.

| **Variables** | AUC | SE | *P*-value | 95% CI |
| --- | --- | --- | --- | --- |
| TyG index | 0.625 | 0.033 | ＜0.001 | 0.560-0.689 |
| TyG index + hypertension + TC | 0.704 | 0.031 | ＜0.001 | 0.644-0.764 |

**Abbreviations:** TC: total cholesterol; TyG index: triglyceride-glucose index; AUC: area under the curve; SE: standard error; OR: odds-ratio; CI: confidence interval.
